# B Cell–Activating Factor Neutralization Aggravates Atherosclerosis

**DOI:** 10.1161/CIRCULATIONAHA.117.032790

**Published:** 2018-11-12

**Authors:** Dimitrios Tsiantoulas, Andrew P. Sage, Laura Göderle, Maria Ozsvar-Kozma, Deirdre Murphy, Florentina Porsch, Gerard Pasterkamp, Jörg Menche, Pascal Schneider, Ziad Mallat, Christoph J. Binder

**Affiliations:** 1 Department of Laboratory Medicine, Medical University of Vienna (D.T., L.G., M.O.-K., F.P., C.J.B.); 2 CeMM Research Center for Molecular Medicine of the Austrian Academy of Sciences (D.T., L.G., M.O.-K., F.P., J.M., C.J.B.); 3 Division of Cardiovascular Medicine, University of Cambridge, UK (A.P.S., D.M., Z.M.).; 4 University Medical Center Utrecht, Netherlands (G.P.).; 5 Department of Biochemistry, University of Lausanne, Epalinges, Switzerland (P.S.).; 6 Institut National de la Santé et de la Recherche Médicale, Paris Cardiovascular Research Center (PARCC), Paris, France (Z.M.).

**Keywords:** atherosclerosis, B-lymphocytes, B-cell activating factor, inflammation, Transmembrane Activator and CAML Interactor Protein

## Abstract

**Background:**

Atherosclerotic cardiovascular disease (heart attacks and strokes) is the major cause of death globally and is caused by the buildup of a plaque in the arterial wall. Genomic data showed that the B cell–activating factor (BAFF) receptor pathway, which is specifically essential for the survival of conventional B lymphocytes (B-2 cells), is a key driver of coronary heart disease. Deletion or antibody-mediated blockade of BAFF receptor ablates B-2 cells and decreases experimental atherosclerosis. Anti-BAFF immunotherapy is approved for treatment of autoimmune systemic lupus erythematosus, and can therefore be expected to limit their associated cardiovascular risk. However, direct effects of anti-BAFF immunotherapy on atherosclerosis remain unknown.

**Methods:**

To investigate the effect of BAFF neutralization in atherosclerosis, the authors treated *Apoe*^−/−^ and *Ldlr*^−/−^ mice with a well-characterized blocking anti-BAFF antibody. Moreover, to investigate the mechanism by which BAFF impacts atherosclerosis, the authors studied atherosclerosis-prone mice that lack the alternative receptor for BAFF: transmembrane activator and calcium modulator and cyclophilin ligand interactor.

**Results:**

The authors demonstrate here that anti-BAFF antibody treatment increased atherosclerosis in mice, despite efficient depletion of mature B-2 cells, suggesting a unique mechanism of action. Indeed, myeloid cell–specific deletion of transmembrane activator and calcium modulator and cyclophilin ligand interactor also results in increased atherosclerosis, while B cell–specific transmembrane activator and calcium modulator and cyclophilin ligand interactor deletion had no effect. Mechanistically, BAFF–transmembrane activator and calcium modulator and cyclophilin ligand interactor signaling represses macrophage IRF7-dependent (but not NF-κB–dependent) Toll-like receptor 9 responses including proatherogenic CXCL10 production.

**Conclusions:**

These data identify a novel B cell–independent anti-inflammatory role for BAFF in atherosclerosis and may have important clinical implications.

## Abstract 2

Supplemental Digital Content is available in the text.

**Clinical Perspective**

**What Is New?**

- Neutralization of B cell–activating factor leads to increased atherosclerotic plaque size and inflammation.
- Deletion of transmembrane activator and calcium modulator and cyclophilin ligand interactor or neutralization of B cell–activating factor increases atherosclerotic plaque size and inflammation.

**What Are the Clinical Implications?**

- Long-term neutralization of B cell–activating factor neutralization may promote atherosclerotic cardiovascular disease.
- Mutations in *Tnfrsf13b* (which encodes transmembrane activator and calcium modulating ligand interactor) that reduced transmembrane activator and calcium modulating ligand interactor signaling in humans, may confer increased cardiovascular risk.

The Global Status Report on Noncommunicable Diseases 2014 issued by the World Health Organization shows that heart attacks and strokes continue to be the major causes of mortality worldwide. A prominent pathology underlying these devastating clinical manifestations is atherosclerosis, a cholesterol-driven chronic inflammatory disease of large- and medium-size arteries that leads to the formation of plaques in the subendothelial space.^1^

B cell and humoral responses are decisively involved in the initiation and progression of atherosclerosis.^2,3^ In addition, genomic and transcriptomic data of participants of the Framingham study indicated proliferation and activation status of B cells to be key factors in determining cardiovascular disease (CVD) risk.^4^ The second most prominent candidate gene in this analysis was *TNFRSF13C*, which encodes 1 of the 3 known receptors for B cell–activating factor (BAFF), BAFF receptor (BAFFR). In mice, disruption of BAFFR signaling results in ablation of, among others, conventional mature B-2 cells (follicular and marginal zone B cells), a very similar phenotype to anti-CD20 antibody (Ab)–treated mice.^5–9^ BAFFR-deficient, anti-BAFFR Ab–treated, and anti-CD20–treated mice display reduced atherosclerosis,^5–9^ suggesting that B-2 cells exhibit proatherogenic properties, and that targeting the BAFFR signaling pathway may be a therapeutic option for combating atherosclerotic CVD.

Alternative immunotherapies target the ligand of BAFFR, BAFF, which exists in multiple forms, including membrane-bound, soluble trimer, and 60-mer forms.^10^ In 2011, the US Food and Drug Administration approved a blocking antibody against soluble BAFF as treatment in systemic lupus erythematosus patients. This anti-BAFF Ab, or a blocking anti-mouse BAFF Ab, leads to apoptosis of mature B cells in both humans^11^ and mice.^12^ However, whether the effect of anti-BAFF Ab treatment on atherosclerosis would recapitulate the results of previous B-2 cell depletion strategies is not clear.

Here, we report the unexpected finding that BAFF neutralization increases atherosclerotic plaque size and complexity despite efficient depletion of mature B-2 cells, and we provide evidence suggesting novel B cell–independent anti-inflammatory properties of BAFF. Consistent with that, we show that the expression of the alternative BAFF-binding receptor transmembrane activator and calcium modulator and cyclophilin ligand interactor (TACI) in myeloid cells limits atherosclerosis, demonstrating novel atheroprotective pathways.

## Methods

We are not able to make the materials used in this study available because of material transfer agreement restrictions.

### Mice, Treatments, and Diets

*Apoe*^−/−^ mice were purchased from Taconic (Denmark); *Ldlr*^−/−^, *Taci*^−/−^ and µMT mice were from The Jackson Laboratories (USA); and *Rag2*^−/−^ mice were purchased from Charles River (Germany). All mice were on C57BL/6 background, and maintained either in the specific pathogen–free facility of the Medical University of Vienna (Austria) or the University of Cambridge (United Kingdom). Female *Apoe*^−/−^ (9–10 weeks old) and male *Ldlr*^−/−^ (12–14 weeks old) mice were injected on day 0, 6, and 14 with 100 µg of either a neutralizing hamster anti-mouse BAFF Ab (clone 10F4; provided by Human Genome Sciences and GlaxoSmithKline) or a control hamster polyclonal immunoglobulin (Ig) G (Jackson Immunoresearch). Preliminary atherosclerosis studies demonstrated no effect of polyclonal IgG on lesion formation versus PBS. From day 14, mice were fed an atherogenic diet (0.2% cholesterol, 21% fat; E15721-347 bought from Ssniff, Germany) for 6 or 8 weeks, and continued to receive the antibody treatment once every 7 to 10 days. *Taci*^−/−^ mice were crossed to *Apoe*^−/−^ mice, and 6-week-old male *Taci*^+/+^, *Taci*^+/-^, and *Taci*^−/−^ mice (on *Apoe*^−/−^ background) were fed an atherogenic diet (21% fat, 0.15% cholesterol; Special Diet Services, United Kingdom) for 6 weeks. Because no significant differences between wild-type and heterozygote mice were observed, data from *Taci*^+/+^ and *Taci*^+/-^ mice were pooled. For bone marrow transplantation experiments, male *Ldlr*^−/−^ mice (8–10 weeks old) were irradiated (10.5 Gy), then reconstituted with 1×10^7^ donor bone marrow cells by IV injection. After a 4-week recovery period, mice were placed on an atherogenic diet for 8 weeks. To achieve B cell–specific—TACI-deficient chimeric mice, the 1×10^7^ cells were made up of 80% µMT (B cell–deficient) with either 20% *Taci*^+/+^ (control group) or 20% *Taci*^−/−^ bone marrow. To create mice with myeloid (non–T cell and non–B cell)–specific deficiency in TACI, the donor bone marrow consisted of 20% C57BL6 wild-type bone marrow and either 80% *Taci*^+/+^*Rag2*^−/−^ (control group) or 80% *Taci*^−/−^*Rag2*^−/−^ bone marrow. All experimental studies were approved by the Animal Ethics Committee of the Medical University of Vienna (BMWF-66.009/0368-WF/V/3b/2014), or have been regulated under the Animals (Scientific Procedures) Act 1986 Amendment Regulations 2012 following ethical review by the University of Cambridge Animal Welfare and Ethical Review Body (PPL PA4BDF775).

### Flow Cytometry

Flow cytometry analysis of splenic and peritoneal B cell subsets was performed as described previously,^6,13^ using directly conjugated antibodies on single cell suspensions of freshly isolated spleens and peritoneal cells. Peripheral blood was diluted with PBS + 2% dextran (Sigma) for at least 30 minutes at 37^°^C to concentrate the erythrocytes at the bottom of the tube. The upper clear phase was collected, and cells were incubated with blocking anti-CD16/32 Ab (clone 93; eBiosciences). Peripheral monocytes and neutrophils were identified by staining with anti-CD11b (allophycocyanin) (clone M1/70; eBiosciences), anti-Ly6C fluorescein isothiocyanate (clone HK1.4; Biolegend), and anti-Ly6G phycoerythrin (clone 1A8; Biolegend). Resident peritoneal and splenic macrophages were stained with an anti-F4/80 Ab conjugated to APC (clone BM8; Biolegend). To quantify TACI expression, cells were stained with anti-TACI PE/APC and isotype control (anti-mouse IgD; clone 11-26c; eBiosciences) where indicated. T regulatory cells were quantified using the Treg detection kit (Miltenyi). Data were acquired on a FACS Calibur or LSRII Fortessa (BD Pharmingen), and were analyzed using FlowJo software 7.6 (Treestar).

### Serum Cholesterol Quantification

Blood was collected from the vena cava (in a MiniCollect Gold cap TUBE; Greiner Bio-One) or the heart at the time of sacrifice. Blood was centrifuged at 1000*g* for 30 minutes at room temperature. Serum total cholesterol was measured in an ISO 15189 accredited medical laboratory under standardized conditions on Beckman Coulter AU5400 (Beckman Coulter) instruments, using the Beckman Coulter OSR6516 reagent, or by colorimetric assay kits bought from Biomerieux (cholesterol).

### Quantification of Atherosclerotic Lesion Size

Atherosclerotic lesion size was evaluated by computer-assisted image analysis using Adobe Photoshop Elements 6.0 and ImageJ software, as previously described.^14^ For the studies with the anti-BAFF Ab, lesion size in the aortic root was quantified in hematoxylin and eosin–stained cross sections (n=9 per mouse) with 50-µm distance that were collected starting with the appearance of all 3 valve leaflets. For studies addressing the role of TACI, atherosclerosis was assessed in the aortic root on serial cryosections by Oil Red O staining, as previously described.^6^

### Immunocytochemistry in Aortic Root Lesions

For quantification of collagen deposition, paraffin-embedded lesions of aortic root were stained with Sirius Red F3B (CI 35782; Sigma-Aldrich). For macrophage content, sections of paraffin-embedded or cryo-sections of aortic root lesions were stained with an anti–MAC-3 (BD Pharmingen) or anti-MOMA2 Ab (Bio-Rad), respectively, as described previously.^13,15^ For CXCL10 quantification, sections were stained with a rabbit anti-CXCL10 Ab (using the commercially available 10H11L3 clone that has been validated in paraffin-embedded spleen sections and in Western blot analysis of recombinant murine CXCL10 by the manufacturer; ThermoFisher). For caspase-3 quantification, we used an anticaspase-3 Ab (Cell Signaling) that specifically detects the cleaved form of caspase-3. Quantification was performed with computer-assisted image analysis using Adobe Photoshop Elements 6.0 and ImageJ software.

### Cytokine and Chemokine Quantification

Murine monocyte chemoattractant protein–1 (MCP-1) and keratinocyte chemoattractant (KC) were quantified in serum samples using the Luminex Technology Procarta Immunoassays (ThermoFisher). Human BAFF was measured in equal total protein lysates from human atherosclerotic plaque extracts from the Athero-Express clinical study.^16,17^

### Bone Marrow–Derived and Peritoneal Macrophage Stimulation

Bone marrow cells were isolated from the femur and tibia of C57BL/6 wild-type mice and were cultivated for 7 days in RPMI1640 containing 10% fetal bovine serum and 1% penicillin/streptomycin (all from Gibco), and supplemented with either 20% conditioned media (from L929 fibroblasts) or 40 ng/mL of recombinant mouse M-CSF (macrophage colony-stimulating factor; Biolegend). Cells were fed on day 3 with the media described above. Resident primary macrophages were isolated from the peritoneum of *Rag1*^−/−^ mice by peritoneal lavage in RPMI1640 containing 1% FBS and 1% penicillin/streptomycin. Peritoneal macrophages were plated in a 96-well (flat-bottom) plate for at least 2 hours before stimulation to allow adherence. To quantify the effect of BAFF in altering TLR9 responsiveness, peritoneal macrophages isolated from *Rag1*^−/−^ mice were stimulated with 0.8 µg/mL BAFF 60-mer (which we had generated previously^18^) in RPMI1640 containing 1% FBS and 1% penicillin/streptomycin overnight, after removing the BAFF-containing media, cells were also stimulated with 1 µmol/L CpG 1826 (Invitrogen) for 4 hours. At the end of the stimulation, cells were lysed in RLT buffer (Peqlab), which is suitable for RNA extraction. For M1 and M2 polarization, bone marrow–derived macrophages were stimulated with either 100 ng/mL interferon-γ (R&D Systems) (M1) or a mixture of 10 ng/mL IL-4 (interleukin-4) and 10 ng/mL of IL-13 (interleukin-13) (both from R&D Systems) for 24 hours. For phagocytosis assays, thymocytes were labeled with CellTracker Green CMFDA (5-chloromethylfluorescein diacetate) Dye (ThermoFisher), and then were incubated with 1 µM of dexamethasone overnight. Then, apoptotic thymocytes (>91% Annexin-V^+^) were added for 3 hours at 5:1 ratio to bone marrow–derived macrophages that had been stimulated overnight with 0.8 µg/mL of BAFF 60-mer. Cells were then stained with anti-F4/80 (allophycocyanin; AbD Serotec) and analyzed by flow cytometry.

### Total RNA Extraction, cDNA Synthesis, and Real-Time Polymerase Chain Reaction Analysis

Total RNA was extracted with the peqGold total RNA kit (Peqlab), or by using TRIzol (Life Technologies) for snap-frozen cells or tissue. cDNA was synthesized using the High Capacity cDNA Reverse Transcription Kit (Applied Biosystems). Quantitative Real-time SYBR Green–based polymerase chain reaction (Peqlab or Eurogentec) was performed on either a Bio-Rad CFX96 real-time system or a Roche Lightcycler. 36B4 was used as reference gene. Data were analyzed using the ΔΔCT method.

### Primer List

1. *Cxcl10* forward: 5-GGATCCCTCTCGCAAGGA-3
2. *Cxcl10* reverse: 5-ATCGTGGCAATGATCTCAACA-3
3. *Cxcl10* forward: 5-GGTCTGAGTGGGACTCAAGG-3
4. *Cxcl10* reverse: 5-GTGGCAATGATCTCAACACG-3
5. *Ifit2* forward: 5-CACCTTCGGTATGGCAACTT-3
6. *Ifit2* reverse: 5-TCAGCACCTGCTTCATCCAA-3
7. *Cxcl1* forward: 5-GCTGGGATTCACCTCAAGAA-3
8. *Cxcl1* reverse: 5-TCTCCGTTACTTGGGGACAC-3
9. *Cxcl2* forward: 5-AGTGAACTGCGCTGTCAATG-3
10. *Cxcl2* reverse: 5-TTCAGGGTCAAGGCAAACTT-3
11. *Taci* forward: 5-GCGCACCTGTACAGACTTC-3
12. *Taci reverse*: 5-GCCTCAATCCTGGACCATG-3
13. *36B4 forward*: 5-AGGGCGACCTGGAAGTCC-3
14. *36B4* reverse: 5-CCCACAATGAAGCATTTTGGA-3
15. *36B4* forward: 5-GTCCTCGTTGGAGTGACATCG-3
16. *36B4* reverse: 5-TAGTTGGACTTCCAGGTCGC-3

### Statistical Analyses

Statistical analyses were performed using Graph Pad Prism 6 for Windows (Graph Pad Software). Experimental groups were compared using an unpaired or paired 2-tailed Student’s or Mann-Whitney test, or one-way ANOVA followed by Tukey’s test as appropriate. Data are presented as mean±SEM. A *P* value of <0.05 was considered significant.

## Results

### Anti-BAFF Treatment Increases Size and Complexity of Atherosclerotic Plaques

**Figure 1.**
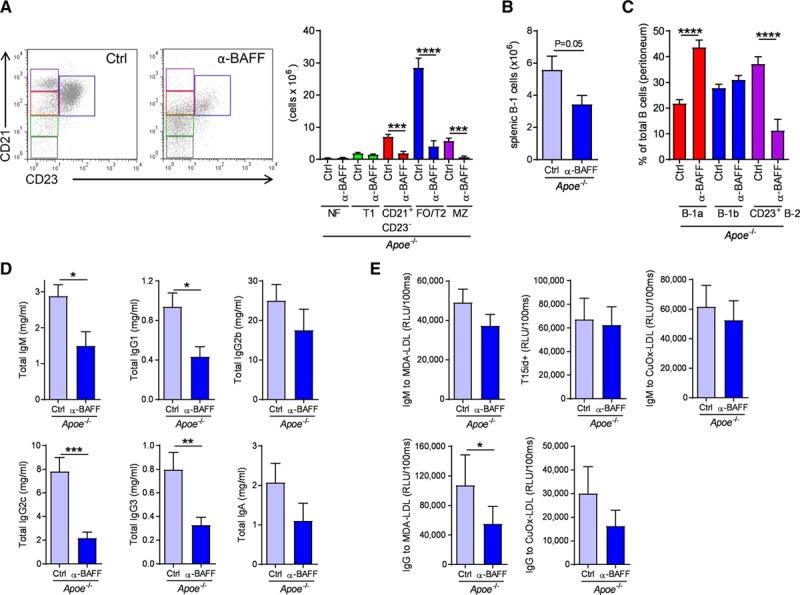
**Anti-B cell–activating factor** (**BAFF) antibody treatment depletes mature B cells and reduces plasma total Ig titers in *Apoe***^***-/-***^** mice.** (**A**) Representative flow cytometry plots and bar graphs show absolute numbers of FO/T2 (blue), MZ (purple), CD21^+^CD23^−^ B cells (red), T1 cells (green), and NF cells (gray), (**B**) absolute numbers of splenic B-1 cells defined as B220^low^IgM^+^CD43^+^, (**C**) frequencies of peritoneal B-1a, B-1b, CD23^+^ B-2 cells, (**D**) total IgM, IgG1, IgG2b, IgG2c, IgG3, and IgA plasma antibody titers, and (**E**) plasma levels of T15id^+^ IgM, MDA^−^, and CuOx-LDL–specific IgG and IgM antibodies. Data are derived from *Apoe*^−/−^ mice treated with a control (Ctrl) or an anti-BAFF neutralizing antibody (α-BAFF) while they were fed an atherogenic diet for 6 weeks (n=8 to 9 mice per group). All results show mean±SEM. *P<0.05, **P<0.01, ***P<0.001, ****P<0.0001 (Mann-Whitney *U* or unpaired Student’s *t* test). CuOx indicates CuSO_4_-oxidized; Ig, immunoglobulin; LDL, low-density lipoprotein; MDA, malondialdehyde-modified; MZ, marginal zone; NF, newly formed; and RLU, relative light units.

**Figure 2.**
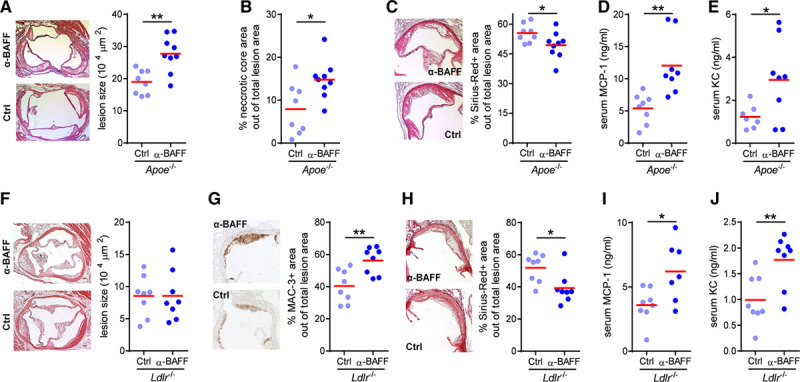
**Anti-B cell–activating factor (BAFF) antibody treatment aggravates atherosclerosis.** (**A**) Representative photomicrographs of hematoxylin and eosin–stained aortic root lesions (50×) and dot plot of the average lesion size in the aortic root expressed as µm^2^ per section (400 µm), (**B**) dot plot of the average necrotic area in the aortic root, (**C**) representative photomicrographs of Sirius Red^+^ area in aortic root plaques, and serum (**D**) KC (keratinocyte chemoattractant) cytokine and (**E**) MCP-1 (monocyte chemoattractant protein-1) chemokine levels of *Apoe*^−/−^ mice fed an atherogenic diet for 6 weeks and injected with a control (Ctrl) or an anti-BAFF neutralizing antibody (n=8 to 9 mice per group). (**F**) Representative photomicrographs of hematoxylin and eosin–stained aortic root lesions (50×) and dot plot of the average lesion size in the aortic origin expressed as µm^2^ per section (400 µm), representative photomicrographs and dot plot of the average (**G**) MAC-3 and (**H**) Sirius Red^+^ area in aortic root plaques, and serum (**I**) KC cytokine and (**J**) MCP-1 chemokine levels of *Ldlr*^−/−^ mice fed an atherogenic diet for 8 weeks and injected with a Ctrl or an anti-BAFF neutralizing antibody (n=7 to 8 mice per group). Dots represent individual mice. All results show mean. *P<0.05, **P<0.01 (Mann-Whitney *U* or unpaired Student’s *t* test). Scale bar: 200 µm.

To investigate the role of BAFF in atherosclerotic plaque development, we treated apolipoprotein E–deficient (*Apoe*^−/−^) mice fed an atherogenic diet for 6 weeks with a hamster antibody neutralizing mouse BAFF (10F4). Anti-BAFF–treated mice displayed similar body weight, plasma total cholesterol, and triglyceride levels compared to control Ab (Ctrl)–treated mice (Table I in the online-only Data Supplement). As expected, anti-BAFF treatment resulted in robust reduction of B-2 cells (Figure 1A) and also in a slight reduction of splenic B-1 cells (Figure 1B). However, peritoneal B-1a and B-1b were proportionally increased or unaffected in anti-BAFF Ab–treated *Apoe*^−/−^ mice, while CD23^+^ B-2 cells were decreased (Figure 1C). Total plasma Igs were decreased in anti-BAFF compared to Ctrl Ab–treated *Apoe*^−/−^ mice (Figure 1D). Furthermore, IgG antibodies—but not IgM—specific for the atherosclerosis-relevant oxidized low-density lipoprotein antigens malondialdehyde-modified low-density lipoprotein and CuSO_4_-oxidized low-density lipoprotein were also decreased in anti-BAFF–treated *Apoe*^−/−^ mice (Figure 1E). Furthermore, B-1a–derived prototypic natural IgM such as T15/E06 also remained unchanged (Figure 1E). Anti-BAFF Ab treatment did not alter splenic total CD4^+^ and T regulatory cell numbers (Figure IA and IB in the online-only Data Supplement). These effects of anti-BAFF treatment on the B cell system are very similar to those described before in anti-CD20–treated and *Baffr*^−/−^ models of atherosclerosis.^2^ Surprisingly, however, anti-BAFF Ab–treated mice developed larger atherosclerotic plaques (Figure 2A), which were further characterized by increased necrotic areas (Figure 2B) and decreased collagen content (Figure 2C), compared to Ctrl Ab–treated mice. Macrophage content (percentage of MAC-3^+^ area out of total lesion area) was not altered (α-BAFF: 28.1±3% versus Ctrl Ab: 33.9±2.5%). BAFF had no direct impact on macrophage efferocytosis in vitro (Figure II in the online-only Data Supplement). However, we found a trend toward increased cleaved caspase-3 content in plaques of anti-BAFF–treated *Apoe*^−/−^ mice compared to Ctrl Ab–treated mice (α-BAFF: 2.1±0.7% versus Ctrl Ab: 0.9±0.4% caspase-3+ area out of total lesion area), suggesting a potential impairment of efferocytosis in vivo. Lesion size in the arch, thoracic, and abdominal aortas (quantified by the en face method) was not different between the 2 groups (α-BAFF: 3.3±0.5% versus Ctrl Ab: 4.6±0.5%). In addition, we found increased levels of proinflammatory KC cytokine and monocyte chemoattractant protein–1 chemokine in the serum of *Apoe*^−/−^ mice that were treated with the anti-BAFF Ab (Figure 2D and 2E), which reflects increased systemic inflammation. We also tested the alternative atherosclerosis-prone model, *Ldlr*^−/−^ mice on a high-fat diet, and treated them with anti-BAFF Ab or Ctrl Ab. Similar to *Apoe*^−/−^ mice, anti-BAFF treatment also resulted in mature B cell depletion and total plasma Ig reduction in *Ldlr*^−/−^ mice (Figure IIIA through IIID in the online-only Data Supplement). Again, anti-BAFF Ab treatment did not change body weight, plasma total cholesterol, and triglyceride levels compared to Ctrl-treated *Ldlr*^−/−^ mice (Table I in the online-only Data Supplement). Atherosclerotic plaque size in the aortic root (Figure 2F) and entire aorta (α-BAFF: 2.4±0.3% *vs.* Ctrl Ab: 2.2±0.4%) was similar between anti-BAFF– and Ctrl–treated *Ldlr*^−/−^ mice, however, anti-BAFF treatment led to increased macrophage lesional content (Figure 2G) and decreased collagen deposition (Figure 2H), indicative of more inflammatory advanced plaques. In line with this, and similar to *Apoe*^−/−^ mice described above, *Ldlr*^−/−^ mice that received anti-BAFF Ab displayed strongly elevated levels of serum KC cytokine and monocyte chemoattractant protein–1 chemokine (Figure 2I and 2J). In addition, anti-BAFF–treated mice displayed increased circulating Ly6C^high^ monocytes and neutrophils (Figure IIIE and IIIF in the online-only Data Supplement). Thus, anti-BAFF treatment promotes inflammation and atherogenesis in atherosclerosis-prone mice.

### BAFF Antagonizes TLR9-IRF7 Responses in Macrophages

**Figure 3.**
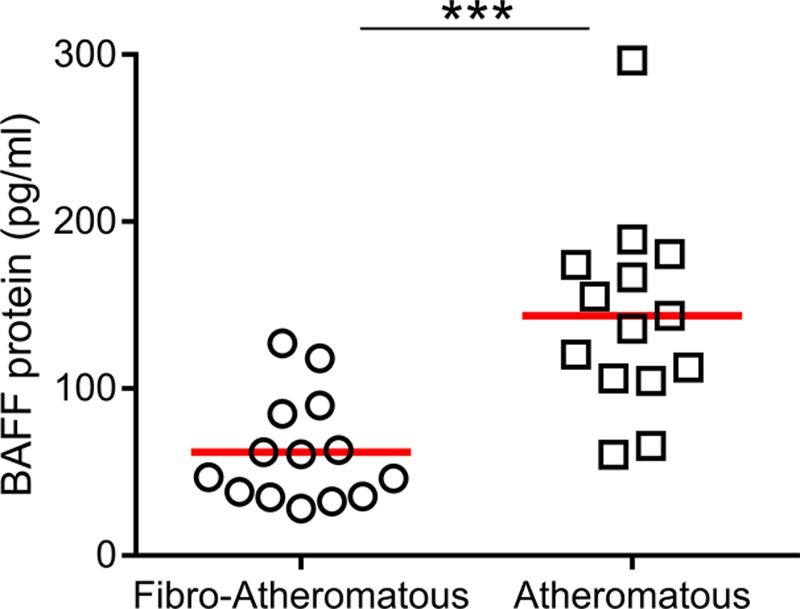
**Increased B cell–activating factor (BAFF) protein content in atheromatous plaques.** Dot plot of the average BAFF protein levels measured by ELISA in equal total protein extracts of human atherosclerotic plaques. Results show mean of n=14 samples per group. ***P<0.001 (unpaired *t* test).

**Figure 4.**
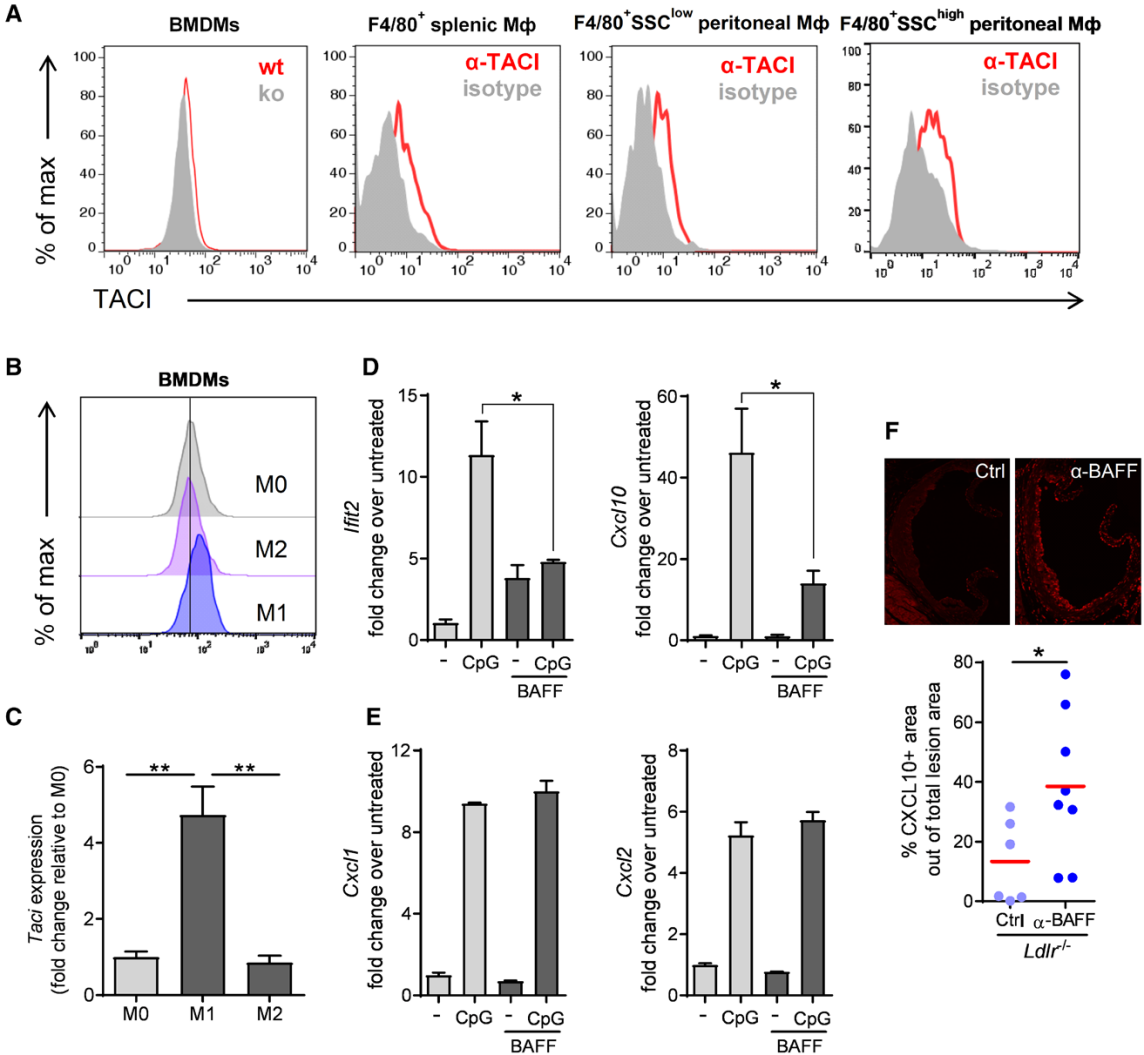
**B cell–activating factor (BAFF) antagonizes TLR9-IRF7 responses in macrophages.** (**A**) Histograms of flow-cytometric analyses of transmembrane activator and calcium modulator and cyclophilin ligand interactor (TACI) expression in bone marrow–derived macrophages (BMDMs) derived from wild-type and *Taci*^−/−^ mice, and F4/80^+^ splenic and peritoneal macrophages isolated from *Rag1*^−/−^ mice. (**B**) Mean fluorescence intensity of surface TACI expression measured by flow cytometry and (**C**) TACI gene expression quantified by real-time polymerase chain reaction (PCR) in BMDMs polarized to M1 or M2 phenotype. Data are representative of (**A**, BMDMs) (**B**) 2 independent experiments and (**A**; F4/80^+^ macrophages) of 3 mice. (**D**) Quantification of (**D**) *Cxcl10* and Ifit2, and (**E**) *Cxcl1* and *Cxcl2* gene expression by real-time PCR in peritoneal resident macrophages (isolated from *Rag1*^−/−^ mice) that were untreated or incubated with BAFF 60-mer (0.8 µg/mL) overnight, and then were stimulated with CpG (1µM) for 4 hours. (**F**) Dot plot shows the CXCL10^+^ area within plaques in the aortic root of *Ldlr* ^−/−^ mice treated with a control (Ctrl) or an anti-BAFF neutralizing antibody (α-BAFF) while they were fed an atherogenic diet for 8 weeks (described in Figure 2F and 2G and Figure III in the online-only Data Supplement; n=6–8 mice per group). (**D** and **E**) Data are representative of at least 2 to 3 independent experiments. All results show mean±SEM. *P<0.05, **P<0.01 (unpaired Student’s *t* test, Mann-Whitney *U* test or 1-way ANOVA followed by Tukey’s test).

The sharply contrasting effects of anti-BAFF treatment, compared to anti-CD20 and anti-BAFFR therapies,^5–9^ led us to explore B cell– and BAFFR-independent effects of BAFF. BAFF is expressed in human plaques, suggesting that BAFF expression may exhibit a local effect in plaques.^19^ We found that human atheromatous plaques contain more BAFF protein compared to fibroatheromatous plaques (Figure 3). These data support the local production of BAFF in plaques. Given the central role of monocytes/macrophages in immune cell regulation of atherosclerosis, we hypothesized that BAFF might affect macrophage responses directly and analyzed the expression of additional BAFF receptors. We found that bone marrow–derived macrophages, resident splenic macrophages, and peritoneal macrophages expressed the TACI receptor (Figure 4A), in line with a recent report.^20^ TACI expression was markedly augmented in M1 polarized macrophages (Figure 4B and 4C), which is the dominant macrophage subset in atherosclerotic lesions.^21^ Heterozygous mutations in the gene encoding TACI are associated with common variable immune deficiency.^22^ Common variable immune deficiency patients carrying such mutations display deregulated TLR9 signaling in B lymphocytes.^23^ These data suggest that BAFF-TACI signaling may be implicated in the regulation of TLR9 signaling. Thus, we investigated the impact of preincubation of peritoneal resident macrophages with TACI-activating recombinant BAFF 60-mer (which efficiently activates the TACI receptor in contrast to BAFF 3-mer, which specifically activates the BAFFR^18^) on TLR9 signaling responses. We found that BAFF specifically dampened TLR9-induced IRF7-dependent expression of interferon-induced with tetratricopeptide repeats 2 (*Ifit2*) and the proatherogenic chemokine *Cxcl10*^24^ (Figure 4D), while it did not affect NF-κB–mediated *Cxcl1* and *Cxcl2* expression (Figure 4E). In agreement with this, mice treated with the anti-BAFF Ab had increased CXCL10 content in atherosclerotic plaques (Figure 4F). CXCL10 has been shown to aggravate experimental atherosclerosis,^24^ and to be an independent prognostic factor for increased CVD risk in humans,^25^ providing a potential mechanism through which BAFF modulates atherosclerotic risk.

### Myeloid Cell–Specific TACI Deletion Increases Atherosclerosis

**Figure 5.**
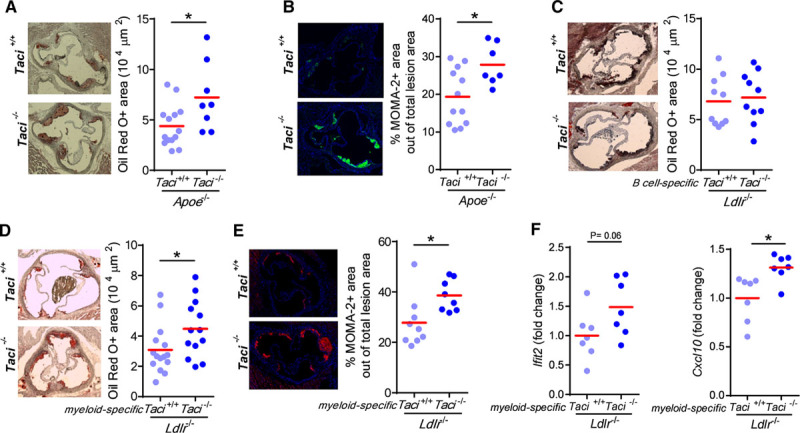
**Myeloid-specific transmembrane activator and calcium modulator and cyclophilin ligand interactor (TACI) signaling confers an atheroprotective effect.** (**A** through **C**) Representative photomicrographs of Oil-Red O stained aortic root lesions and dot plot of the average lesion size in the aortic origin expressed as µm^2^ per section, and (**B**) representative photomicrographs and dot plot of the average MOMA-2^+^ area in aortic root plaques of the of (**A**) *Apoe*^−/−^*Taci*^+/+^ and *Apoe*^−/−^*Taci*^−/−^ mice fed an atherogenic diet for 6 weeks (n=7 to 14 per group). (**C** and **D**) Representative photomicrographs of Oil-Red O stained aortic root lesions and dot plot of the average lesion size in the aortic origin expressed as µm^2^ per section of lethally irradiated *Ldlr*^−/−^ mice transplanted with a mixture of bone marrow cells made up of (**C**) 80% µMT (B cell–deficient), with either 20% *Taci*^+/+^ (control group) or 20% *Taci*^−/−^ bone marrow (n=10 per group) or (**D**) of 20% C57BL/6 wild-type bone marrow and either 80% *Taci*^+/+^*Rag2*^−/−^ (control group) or 80% *Taci*^−/−^*Rag2*^−/−^ bone marrow (n=13 to 15 per group), and were fed an atherogenic diet for 8 weeks. (**E**) Representative photomicrographs and dot plot of the average MOMA-2^+^ area in aortic root plaques of the *Ldlr*^−/−^ mice described in (**D**) (n=8 to 9 per group). (**F**) Quantification of *Cxcl10* and *Ifit2* gene expression in the spleen of the *Ldlr*^−/−^ mice described in (**D**), by real-time polymerase chain reaction (n=7 per group). Dots represent individual mice. All results show mean. *P<0.05 (Mann-Whitney *U* or unpaired Student’s *t* test).

Consistent with TACI-mediated atheroprotective effects of BAFF, we found that *Apoe*^−/−^ *Taci*^−/−^ mice fed an atherogenic diet developed bigger atherosclerotic plaques, with more macrophages, compared to *Apoe*^−/−^ *Taci*^+/+^ controls (Figure 5A and 5B). To identify the cell compartment that is responsible for the atheroprotective effect of TACI, we studied atherosclerotic mice that lack TACI, specifically either in B cells (induced on transplantation of mixed *Taci*^−/−^ and µMT bone marrow cells into lethally irradiated recipients; Figure IVA in online-only Data Supplement) or myeloid cells (induced on transplantation of mixed *Taci*^−/−^ *Rag2*^−/−^ and wild-type bone marrow cells into lethally irradiated *Ldlr*^−/−^ recipients; Figure IVB in online-only Data Supplement). In agreement with a B cell–independent role for BAFF in atherosclerosis, we found that atherogenic diet–fed *Ldlr*^−/−^ mice with B cell–specific deletion of TACI, which did not alter splenic B cell numbers (Figure VA in online-only Data Supplement), or total cholesterol levels (Table I in online-only Data Supplement), did not impact atherosclerosis (Figure 5C). On the contrary, myeloid cell–specific deletion of TACI led to increased atherosclerosis (Figure 5D). In addition, and similar to the effect of global TACI deficiency in *Apoe*^−/−^ mice, myeloid cell–specific deletion of TACI in *Ldlr*^−/−^ mice resulted in enhanced macrophage content (Figure 5E). Myeloid cell–specific TACI deletion did not change splenic B cells (Figure VB in online-only Data Supplement) or total plasma cholesterol levels (Table I in online-only Data Supplement). Furthermore, myeloid cell–specific TACI deletion resulted in increased expression of *Cxcl10* and *Ifit2* expression in the spleens of atherosclerotic mice in vivo (Figure 5F), mirroring the in vitro effects of BAFF-TACI signaling in regulating the TLR9-IRF7 pathway in macrophages.

## Discussion

The aim of the present study was to investigate the effect of BAFF neutralization in the development of atherosclerosis. Our data show that treatment with neutralizing anti-BAFF Ab detrimentally affects lesion development in 2 mouse models of atherosclerosis, without affecting lipid metabolism. Anti-BAFF treatment induced characteristics of advanced atherosclerosis in both *Apoe*^−/−^ and *Ldlr*^−/−^ mice, such as decreased collagen content in plaques and increased levels of circulating proinflammatory KC cytokine and monocyte chemoattractant protein–1 chemokine. However, we found it increased lesion size only in *Apoe*^−/−^, and not in *Ldlr*^−/−^ mice. In our study, *Apoe*^−/−^ mice were fed an atherogenic diet for 6 weeks and displayed bigger lesions with more inflammatory cells compared to *Ldlr*^−/−^ mice. Along this line, we propose that the apparent differences of BAFF blockade in the 2 models are linked to the different stage of atherosclerotic plaque development in *Apoe*^−/−^ and *Ldlr*^−/−^ mice under these settings, rather than distinct mechanisms. Others recently reported that mice overexpressing both soluble and membrane-bound forms of BAFF (BAFF-transgenic)^10^ exhibited decreased atherosclerosis in association with a profound (approximately 50%) reduction in plasma total cholesterol levels that are likely responsible for the effect observed in these mice.^26^ Moreover, although informative, we believe that these BAFF-transgenic mice, which express at least 10 and up to 400 times higher levels of BAFF (ie, higher than what can be measured in any pathophysiological conditions in mice or humans), mostly reflect a pharmacological rather than a pathophysiological setting. Mice with more physiologically relevant elevations of circulating BAFF, as is the case in B cell–deficient settings,^27^ do not show any alterations of plasma cholesterol levels. Furthermore, we are not aware of any report of reduced plasma cholesterol levels in patients with high BAFF levels.

The atheroprotective effect of disrupted BAFF-BAFFR signaling has been attributed to depletion of conventional proatherogenic B-2 cells.^8,9^ We show here that soluble BAFF also has the capacity to affect atherosclerosis beyond changes in BAFFR signaling and B cell immunity, as atherosclerotic mice treated with the anti-BAFF neutralizing Ab display a similar reduction in mature B cells and circulating Ig levels, yet they develop increased atherosclerosis.

Notably, BAFFR-deficient mice also display robustly increased circulating BAFF levels.^28^ Thus, it is even possible that the increased soluble BAFF levels in BAFFR-deficient^5,6^ or B cell–depleted^8,9^ mice might also contribute to the atheroprotective effect in these settings. In addition to BAFFR, BAFF also binds to B cell maturation antigen and TACI,^10^ which we found to be highly expressed in M1-like polarized macrophages. However, BAFF—in contrast to TACI—displays a particularly low affinity toward B cell maturation antigen, and thus we hypothesized that BAFF mediates its atheroprotective effect via the TACI receptor. Consistent with this, we show that global as well as myeloid-specific TACI deletion increases atherosclerosis and macrophage lesional content, which suggests—in analogy to the anti-BAFF studies—that TACI impacts atherosclerosis via similar mechanisms in both models. The increased atherosclerosis in this study was associated with enhanced inflammatory markers, both locally and systemically. In addition, anti-BAFF treatment increased Ly6C^high^ peripheral monocytes. Taken together, these data suggest that BAFF can affect monocyte/macrophage responses directly. Consistent with this, circulating CD14^+^CD16^+^ monocytes, which are a human equivalent to Ly6C^high^ monocytes and have been shown to positively associate with human coronary atherosclerosis,^29^ are decreased in individuals with a genetic variant of TNFSF13B (which encodes BAFF and leads to increased soluble BAFF levels).^30^ Peritoneal macrophages express BAFFR mRNA, though no protein was detected on the cell surface.^20^ However, global BAFFR deficiency resulted in a similar atheroprotective effect to B cell–specific BAFFR deletion,^6^ which suggests that lack of BAFFR signaling in macrophages is unlikely to be involved in the proatherogenic effect of anti-BAFF treatment. In addition to BAFF, TACI is also bound by the alternative ligand APRIL (A Proliferation Inducing Ligand).^10^ However, a recent study showed that APRIL overexpression in *Apoe*^−/−^ mice does not affect atherosclerotic plaque size.^31^ Thus, it is unlikely that increased APRIL signaling in BAFFR-deficient^5,6^ or B cell–depleted^8,9^ mice is responsible for the atheroprotective effects observed.

Anti-BAFF therapy is now considered to be a main line of treatment in a subset of systemic lupus erythematosus patients, who exhibit a strong predisposition to premature atherosclerosis and heart attacks.^32,33^ The protective effect of BAFF inhibition in systemic lupus erythematosus has been attributed to its B cell–depleting effect. However, rituximab treatment (anti-CD20 Ab), which provides a more efficient and rapid B cell depletion, did not show any clinical benefit in systemic lupus erythematosus patients in 2 double-blind clinical trials.^34^ Therefore, it is likely that long-term BAFF neutralization in humans triggers additional effects (beyond B cell depletion and plasma auto-Ab reduction) that have important pathophysiological consequences. This is particularly interesting as we show that human atheromatous plaques, which typically contain a higher number of inflammatory cells, have significantly increased levels of BAFF protein compared to fibroatheromatous plaques. Therefore, BAFF may affect macrophage responses locally, eg, by dampening proinflammatory responses such as CXCL10 expression, as we have shown, and/or preventing cell death in lesions. Indeed, it has been shown that stimulation of human monocytes with BAFF promotes cell survival.^35^

The role of TLR9 responses in atherosclerosis remains inconclusive. Our data suggest that preferential engagement of the IRF7 or NF-κB pathway may determine the effect of TLR9 stimulation in macrophages on plaque formation.^36,37^ Koulis et al have shown that TLR9 deletion promoted atherosclerosis, and that treatment with the TLR9 ligand CpG ODN 1668 reduced atherosclerosis.^36^ On the other hand, Krogmann et al demonstrated that treatment of atherosclerotic *Apoe*^−/−^ mice with CpG ODN 1826 enhanced atherosclerosis.^37^ Our data support a proatherogenic role of the TLR9-IRF7 pathway. In line with this, it has been shown that treatment with CpG ODN 1585 increased CXCL10 production and enhanced atherosclerosis in *Apoe*^−/−^ mice.^38^ We show that the BAFF-TACI signaling pathway preferentially represses IRF7-mediated responses in TLR9-stimulated macrophages, leading to decreased C*xcl10* expression. Notably, it has been reported that macrophages from TACI-deficient mice acquire an M2-like phenotype and display lower expression levels of TLR4 and TLR9.^20^ These data suggest that TACI signaling might enhance the proinflammatory properties of macrophages (eg, in situations of hypercholesterolemia). Nevertheless, our data indicate that atherosclerotic mice lacking TACI signaling in myeloid cells express increased amounts of typical TLR9-induced genes, such as *Ifit2* and *Cxcl10* transcripts, in their spleens and developed increased atherosclerosis. Furthermore, anti-BAFF treatment lead to increased CXCL10 content in plaques. CXCL10 has been shown to exhibit proatherogenic properties in experimental atherosclerosis,^24^ and to be an independent prognostic factor for increased CVD risk in humans.^25^ Therefore, we propose that BAFF-TACI signaling represses proatherogenic responses in macrophages under hypercholesterolemic conditions.

While no accelerated CVD complications have been reported on anti-BAFF therapy so far, our findings introduce a new perspective with respect to the potential cardiovascular hazard that may be associated with the blockade of BAFF in chronic cardiovascular inflammatory settings, in contrast to acute cardiovascular inflammation.^39^ In conclusion, there is a need for more refined therapeutic approaches targeting the BAFF pathway.

## Sources of Funding

This work was supported by grants of the Austrian Science Fund (SFB F54), the European Union (FP7 VIA), the British Heart Foundation, and the European Research Council. Dr Schneider is supported by grants from the Swiss National Science Foundation.

## Disclosures

Dr Schneider is supported by a research grant from EMD Serono, a subsidiary of Merck, KGaA.

## Supplementary Material
