# Supplementary material for: B Cell–Activating Factor Neutralization Aggravates Atherosclerosis
Source: Circulation. 2018 Nov 12;138(20):2263–73. doi: 10.1161/CIRCULATIONAHA.117.032790 (PMC6181204; doi:10.1161/CIRCULATIONAHA.117.032790)
Supplement: Supplementary file 1 [file cir-138-2263-s001.pdf]

SUPPLEMENTAL MATERIAL

Supplement Table 1

Study	experimental groups	final body weight (g)	total cholesterol (mg/dL)
anti-BAFF / $Apoe^{-/-}$	anti-BAFF Ab	33.8 ±1.5	1551 ±92
	Ctrl Ab	34.6 ±2.2	1679 ±45
anti-BAFF / $Ldlr^{-/-}$	anti-BAFF Ab	32.2 ±0.9	1680 ±140
	Ctrl Ab	32.3 ±0.7	1727 ±112
$Apoe^{-/-}$ Taci	$Taci^{-/-}$	29.8 ±0.8	949 ±46
	$Taci^{+/+}$	31.1 ±0.8	933 ±39
$Ldlr^{-/-}$Taci (myeloid-specific)	$Taci^{-/-}$	31.1 ±0.9**	1056 ±128
	$Taci^{+/+}$	35.3 ±1.1	960 ±74
$Ldlr^{-/-}$ Taci (B cell-specific)	$Taci^{-/-}$	30.2 ±0.7	840 ±47
	$Taci^{+/+}$	30.5 ±0.7	789 ±33

Supplement Table 1. Whole body weight and serum total cholesterol. Data show body weights and total cholesterol levels determined at the end of each experiment. All results are represented as mean ± SEM.

Supplement Figure 1

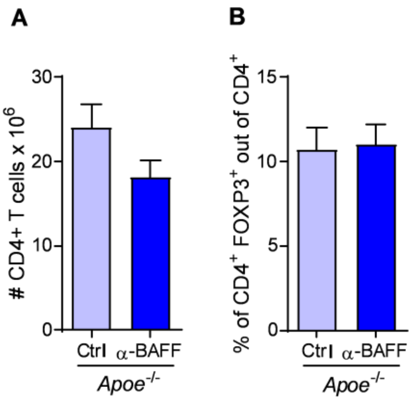

Supplement Figure 1. Anti-BAFF antibody treatment does not alter the numbers of splenic CD4$^+$ T cells and Tregs. Bar graphs show absolute numbers of (**A**) CD4$^+$ T cells (**B**) Treg (CD4$^+$ FOXP3$^+$) frequency within CD4$^+$ T cells in the spleen of *Apoe* $^{-/-}$ mice treated with a control (Ctrl) or an anti-BAFF neutralizing antibody (α-BAFF) while they were fed an atherogenic diet for 8 weeks (n=8-9 mice per group). All results show mean ± SEM.

Supplement Figure 2

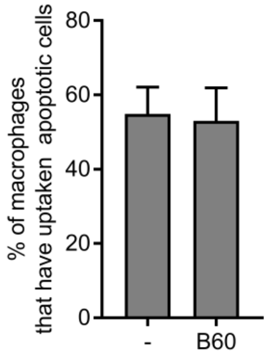

Supplement Figure 2. BAFF stimulation does not alter the phagocytic capacity of macrophages in vitro. Bar graph shows the mean derived from the average of quadruplicates of three independent experiments (paired t test).

Supplement Figure 3

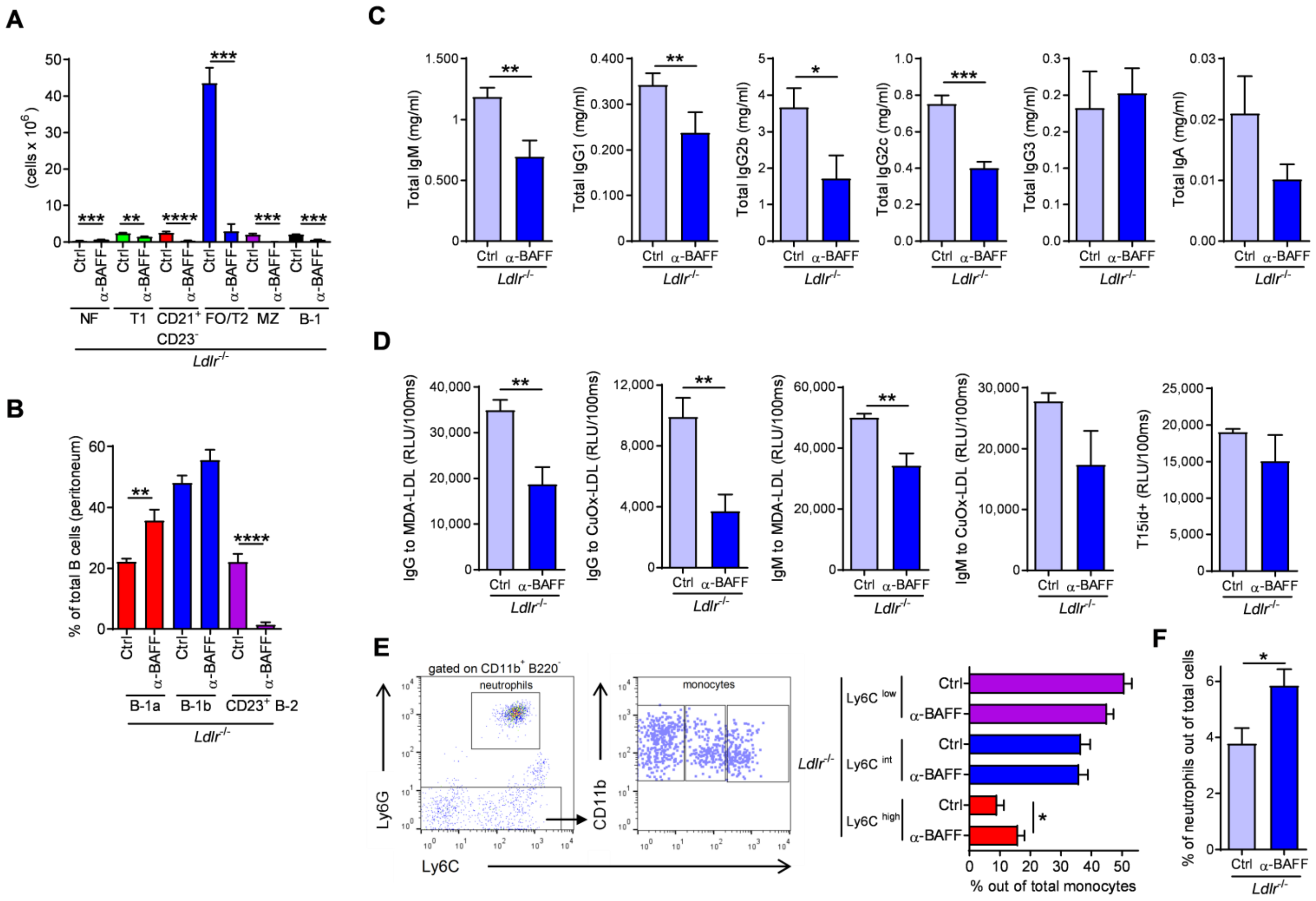

Supplement Figure 3. Anti-BAFF antibody treatment depletes mature B cells and reduces total Ig titers in plasma in *Ldlr*$^{-/-}$ mice. (**A**) Bar graphs show absolute numbers of FO/T2 (blue), MZ (purple), CD21^{+}CD23^{-} B cells (red), T1 (green), NF (grey) cells and B-1 cells (defined as B220^{low}IgM^{+}CD43^{+}), (**B**) frequencies of peritoneal B-1a, B-1b, CD23^{+} B-2 cells, (**C**) Total IgM, IgG1, IgG2b, IgG2c, IgG3 and IgA plasma antibody titers, (**D**) plasma levels of T15id+ IgM, MDA- and CuOx-LDL specific IgG and IgM antibodies, (**E**) representative flow cytometry gating strategy (left) and frequencies of circulating Ly6C^{high}, Ly6C^{int} and Ly6C^{low} monocytes (right) and (**F**) frequencies of neutrophils in peripheral blood. Data are derived from *Ldlr* $^{-/-}$ mice treated with a control (Ctrl) or an anti-BAFF neutralizing

antibody (α-BAFF) while they were fed an atherogenic diet for 8 weeks (n=8 mice per group). All results show mean ± SEM. *P<0.05, **P<0.01, ***P<0.001, ****P<0.0001 (Mann-Whitney *U* or unpaired Student *t* test).

Supplement Figure 4

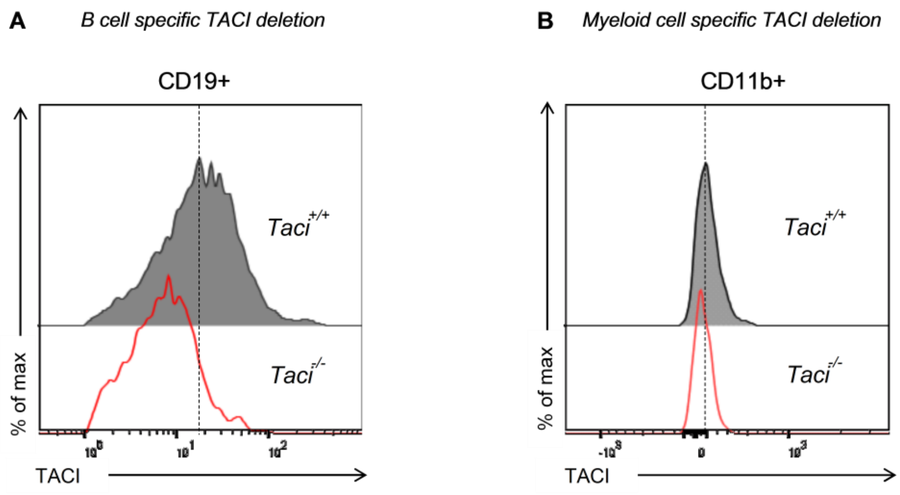

Supplement Figure 4. Evaluation of B cell or myeloid cell specific TACI deletion of atherosclerosis studies shown in Figure 2. (**A**; B cell specific TACI deletion) Histograms show mean fluorescence intensity (MFI) of surface TACI expression measured by flow cytometry in B cells (CD19$^+$) of lethally irradiated *Ldlr*$^{-/-}$ mice transplanted with a mixture of 80% µMT (B cell deficient) with either 20% *Taci*$^{+/+}$ (*Taci*$^{+/+}$) or 20% *Taci*$^{-/-}$ bone marrow (*Taci*$^{-/-}$). (**B;** myeloid cell specific TACI deletion) Histograms show MFI of surface TACI expression measured by flow cytometry in myeloid cells (CD11b$^+$) of lethally irradiated *Ldlr*$^{-/-}$ mice transplanted with a mixture of 20% C57BL/6 WT bone marrow and either 80% *Taci*$^{+/+}$*Rag2*$^{-/-}$ (*Taci*$^{+/+}$) or 80% *Taci*$^{-/-}$*Rag2*$^{-/-}$ bone marrow (*Taci*$^{-/-}$). All mice were fed an atherogenic diet for 8 weeks.

Supplement Figure 5

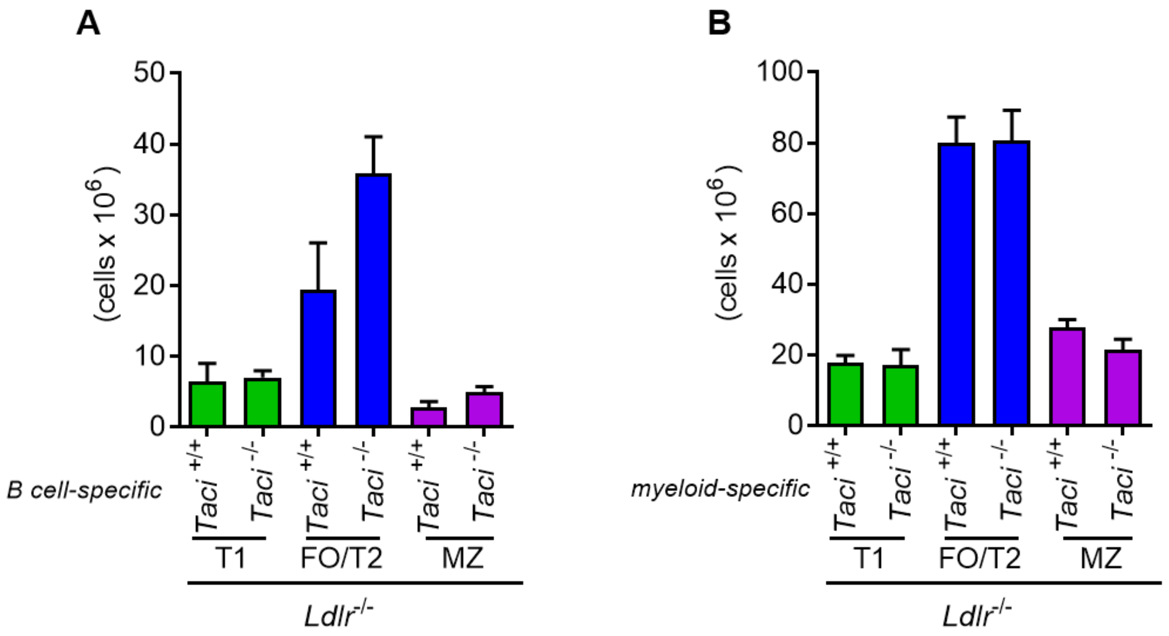

Supplement Figure 5. TACI signaling does not alter splenic B cell numbers in atherosclerosis-prone mice. Bar graphs show absolute numbers of FO/T2 (blue), MZ (purple) and T1 (green) in the spleens of lethally irradiated *Ldlr*$^{-/-}$ mice transplanted with a mixture of bone marrow cells made up of (**B**) 80% µMT (B cell deficient) with either 20% *Taci*$^{+/+}$ (control group) or 20% *Taci*$^{-/-}$ bone marrow (n=10 per group) or (**C**) of 20% C57BL6 WT bone marrow and either 80% *Taci*$^{+/+}$*Rag2*$^{-/-}$ (control group) or 80% *Taci*$^{-/-}$*Rag2*$^{-/-}$ bone marrow (n=13-15 per group), and were fed an atherogenic diet for 8 weeks.
